# Malaria infection prevalence and diagnostic performance of the Abbott Bioline Malaria Ag P.f/Pan rapid test in rural populations of Central Cameroon

**DOI:** 10.1038/s41598-025-13688-8

**Published:** 2025-07-31

**Authors:** Viviane Ongbassomben Missoup, Pierre Fongho Suh, Carine Nkodo Ndjebakal, Darus Tagne, Yacouba Poumachu, Steve Joko, Alima Kouamendjouo Djilla, Flobert Njiokou, Wilfred Mbacham, Charles Wondji, Cyrille Ndo

**Affiliations:** 1 Faculty of Sciences, The University of Yaoundé I, P.O. Box 812, Yaoundé, Cameroon; 2 Department of Parasitology and Microbiology, Centre for Research in Infectious Diseases (CRID), P.O Box 13591, Yaoundé, Cameroon; 3 Faculty of Medicine and Pharmaceutical Sciences, The University of Douala, P.O. Box 2701, Douala, Cameroun; 4 Institut de Recherche de Yaoundé (IRY), Organisation de Coordination pour la lutte contre les Endémies en Afrique Centrale (OCEAC), P.O Box 288, Yaoundé, Cameroon; 5 Vector group, Liverpool School of Tropical Medicine, Pembroke Place, Liverpool, L3 5QA UK

**Keywords:** Malaria, Abbott Bioline Malaria ag pf/pan rapid diagnostic test, Central-Cameroon, Biological techniques, Microbiology, Diseases

## Abstract

In Cameroon, the management of uncomplicated malaria cases in the communities and in low-resource health facilities rely on the use of reliable rapid diagnostic tests (RDTs). This work was undertaken to determine the trend in human malaria infection in rural settings of Central Cameroon and assess the diagnostic performance of Abbott Bioline Malaria Ag P.f/Pan RDT recommended by the National Malaria Control Programme (NMCP). Cross-sectional surveys were conducted in March 2022 and May 2024. *Plasmodium* infection was detected using RDT and microscopy techniques, and with real-time PCR for validation of discordant results. Sensitivity (Se), specificity (Sp), positive and negative predictive values, positive and negative likelihood ratios (LR + and LR-), accuracy and agreement were calculated to assess the performance of the RDT. *Plasmodium* infection prevalence was 64.9% and 71.7% by microscopy and RDT, respectively, *Plasmodium falciparum* being the predominant species (microscopy:97.48%). With microscopy as reference, the RDT showed high sensitivity (Se:93.37%; CI:91.15%-95.77%) and low specificity (Sp: 68.50%; CI:62.40%-74.17%) for the detection of *P. falciparum* infection. The positive and negative predictive values were respectively 84.43% (CI:81.01%-87.46%) and 85.71% (CI:80.13%-90.22%). The RDT showed a small positive likelihood ratio (LR + = 2.96; CI:2.47-3.5734), a good negative likelihood ratio (LR-=0.09; CI:0.06–0.13) and moderate agreement (k = 0.652; CI:0.593–0.710; *P* < 0.001) with microscopy. The RDT showed higher sensitivity (81.48% vs. 48.14%), accuracy (0.75 and vs. 0.50), and agreement (AC1 = 0.715 vs. 0.371) than microscopy. The Abbott Bioline Malaria Ag P.f/Pan RDT demonstrated a high level of agreement with the most sensitive qPCR technique compared to microscopy. These findings further support its use as a reliable malaria diagnostic tool in the highly endemic setting of Central Cameroon.

## Introduction

Malaria is a potentially deadly disease caused by unicellular protozoan parasites of the genus *Plasmodium,* which are transmitted to humans through the bites of female *Anopheles* mosquitoes^1^. In Cameroon, malaria remains a major public health and development issue. The country belongs to the block of the eleven African countries that carry approximately 70% of the global burden of malaria^2^. The disease is endemic throughout the country, with year-round transmission in humid savanna and forested areas of the Southern part, while it is seasonal in the Sahelian North. Four of the five *Plasmodium* species that are capable to infect human are present, but most of the cases across the country are due to *P. falciparum*^3^.

Cameroon has adopted the *Global Malaria Technical Strategy 2016–2030* and its vision to lead to a malaria-free world by 2030^4,5^. In this vein, the country continues to invest remarkable efforts for disease prevention, notably through the scaling-up of high-impact interventions. These include universal distribution of Long-Lasting Insecticidal-Nets (LLINs) during mass campaigns and as a routine through health facilities targeting pregnant women and children under 5 years. In addition, intermittent preventive treatment (IPT) for pregnant women and seasonal Malaria Chemoprevention (SMC) in children aged between 3 and 59 months in the North, are other priority interventions that are implemented for the prevention of malaria. The use of the RTSS vaccine was introduced in 42 health districts nationwide for all children aged 6 to 24 months old in January 2024, with an overall monthly immunization coverage of 37%^6,7^.

Besides prevention strategies deployed, the management of cases is among central pillars for malaria control. The World Health Organization (WHO) recommends parasitological confirmation by microscopy and/or rapid diagnostic tests (RDTs) for all patients suspected of having malaria, before initiating any treatment^8^. While rapid tests and microscopy are the most commonly used methods for diagnosing malaria, their sensitivity is somewhat limited. In contrast, molecular analysis offers excellent sensitivity (0.004–30 parasites/µl of blood), though it is more expensive and requires specialized technical equipment and trained personnel^9,10^. In rural areas of Cameroon, where the majority of malaria transmission occurs, the conditions for the implementation of microscopy are usually not met. These areas suffer from a lack of an appropriate technical platform including microscope and reagents, poor electrical power supply and shortage of skilled technicians to prepare and read thick blood films^6^. In addition, the time taken to perform microscopic diagnosis is fairly long, and this can be detrimental to the management of malaria cases in emergencies^11^.

Due to difficulties associated with the implementation of quality microscopy, the National Malaria Control Programme (NMCP) recommends for its 2024–2028 strategic plan to expand access to the diagnosis of malaria using only RDTs, particularly in health facilities having low resources, as well as for case management of uncomplicated malaria by community health workers^4^. The adoption of such a strategy requires the use of reliable RDTs that can promptly and accurately detect *Plasmodium* infections. National policies recommend the use of RDTs that detect the Histidine Rich Protein 2 of *P. falciparum*, which is the major malaria species across the country. In addition, RDTs should also target the Pan Lactate Dehydrogenase (PfHRP2/Pan) proteins, which is common to all *Plasmodium* species, allowing simultaneous diagnosis of non-*falciparum* infections.

The performance of this tool is unfortunately being challenged by several factors including low parasite densities observed notably in cases of asymptomatic malaria and the apparition of deletion in *Pfhrp2/3* genes encoding the HRP2/3 protein, causing false negative results. This situation could have consequences on management decision, especially in rural areas, where most cases of malaria occur. The present study aimed to evaluate the diagnosis performance of the Abbott Bioline Malaria Ag P.f/Pan rapid diagnostic test (Abbott Diagnostic Inc, Korea) which is widely distributed throughout the country and recommended by the NMCP for malaria diagnosis, at both community and health facility levels.

## Methodology

### Study sites

Cross-sectional surveys were carried out in two rural health districts of the Central region of Cameroon, from March 2022 to May 2024. The Obala and Okola districts are located 62 km and 25 km from Yaoundé, respectively, and are more than 60 km apart. The study localities are located within the deep-evergreen equatorial forest zone and have a Guinean-type climate characterized by two dry seasons (mid-June to mid-August; mid-November to mid-March) and two rainy seasons (mid-March to mid-June; mid-August to mid-November). The average annual rainfall there is 1577 mm, and the average annual temperature is 25°C. As they are located in the Centre region, they are considered to belong to a holoendemic stratum with high and perennial malaria parasite transmission^12^. In Obala, the survey was conducted at Emana-Benyada village (4°15’35.8’’N, 11° 36’41.9’’E), while in Okola investigations were carried out at Nkol-fep (3˚59̛ 9ˮN, 11˚ 23̛ 35ˮ E) and Nkol-odou (03˚57. 8462̛ N and 011˚21.6988̛ E) villages as well as in five surrounding schools namely Ebougsi, Lengon, Elig-Onana, Mva’a and Zamengoue Primary Schools.

### Study population

- ***Inclusion***,*** exclusion and non-inclusion criteria***

In the community, any individual symptomatic or not, who had resided in the villages of Emana-Benyada, Nkol-feb and Nkol-odou for at least 14 days and had signed an informed consent form was included. In schools, symptomatic and asymptomatic children aged between 6 and 14 years, whose parents or legal guardians had signed a consent form were enrolled. In the community and at local schools, individuals who had received antimalarial treatment in the 30 days prior to the study were non included, to avoid false positive RDT results due to possible persistence of the *P. falciparum* histidine-rich protein 2 (PfHRP2) in the bloodstream. Similarly, anyone showing signs of severe malaria and requiring emergency treatment was not included. Moreover, participants for whom results of both RDT and microscopy tests were not available were excluded from the analysis.

- ***Sampling method and sample size***

Participants were recruited using simple random sampling method and each of them was examined once. The minimum sample size calculated based on the Lorentz formula was 277 participants.

n = Z^2^*P* (1-P)/i^2^, with a significance level of 5% (i), confidence level (Z) of 95% and *P* = 76.41% (the malaria prevalence in the Centre region of Cameroon reported in 2021 by the NMCP).

### Administrative procedures and ethical considerations

Prior to the beginning of each study period, an ethical clearance (N°2021/11/1404CE/CNERSH/SP and N°2023/04/1531CE/CNERSH/SP) was granted by the National Ethics Committee for Research in Human Health (NECRH). Thereafter, administrative authorizations were delivered by the Cameroon’s Ministry of Public Health and the regional delegate for basic education. Written informed consents were obtained from all participants ≥ 18 years, and from parents or legal guardians for those < 18 years. All research was performed in accordance with relevant guidelines/regulations. Participants who were found positive for malaria received free antimalarial treatment according to national guidelines for the management of malaria.

### Data collection

A semi-structured questionnaire was administered to participants above 15 years and to parents or legal guardians of children aged less than 15 years to gather their socio-demographic information (age, gender, village), the time they have spent in the village and their malaria history (malaria infection within four weeks prior to the study and use of antimalarial drugs). Additionally, participant’s body temperature was recorded using an infrared thermometer (Rapid Electronic) and values of 38 °C or above were indication of fever.

### Definition of terms

Definitions of key variables of this study were as follow:

True positive (TP): sample both positive by RDT microscopy.

True negative (TN): sample negative by RDTs and negative by microscopy.

False positive (FP): sample positive by RDTs but negative by microscopy.

False negative (FN): sample negative by RDT but positive by microscopy.

Sensitivity: proportion of true positive tests out of all participants with *Plasmodium* infection.

Specificity: percentage of true negatives out of all subjects who do not have *Plasmodium* infection.

Positive predictive value: proportion of true positive among all the positive results.

Negative predictive value: proportion of true negative among all the negative results.

Positive likelihood ratio (LR+): likelihood that someone with *Plasmodium* infection had a positive test compared to someone without the infection.

Negative likelihood ratio (LH-): likelihood that someone with *Plasmodium* infection had a negative test compared to someone without the infection.

Accuracy: proportion of true results given by a test.

Agreement: degree of concordance between two or more sets of measurements.

### Diagnosis of malaria parasites by RDT

The Abbott Bioline Malaria Ag P.f/Pan kits were used to detect malaria infection. The test targets the HRP2 (*histidine-rich protein 2*) protein specific to *P. falciparum* and the Lactate dehydrogenase which is common to *Plasmodium* species^19^. The test procedure was done according to the manufacturer’s instructions^13,14^. Briefly, the test device was removed from the foil, labelled and placed on a table with a flat and dry surface. Participant’s fingertip was cleaned with a disposable alcohol swab, then the lateral side of the finger was picked with the sterile lancet provided. The first drop of blood was wiped away with a cotton ball and the second one was collected by dipping the circular end of an inverted capillary pipette (5 µl) into the blood specimen. The collected blood was then transferred into the round specimen well, and four drops of assay diluent were dispensed vertically into the square assay diluent well. The test results were read and interpretated 15 to 30 min after adding assay diluent, by two independent technicians. One cassette was used for each participant and all tests that produced invalid results were repeated.

### Detection of malaria parasites by microscopy

Thick and thin blood smears were prepared using freshly collected blood from a finger prick and air-dried. The thin smear was fixed with methanol for two minutes, then the two preparations were stained with freshly prepared 10% Giemsa solution for 25–30 min, according to WHO standard^15^. Stained slides were washed, air-dried and observed using x100 objective (oil immersion) under the light microscope, by two experienced microscopists. The slides were considered negative when no malaria parasite was observed after reading 100 power fields. For positive samples, malaria parasites were counted in thick blood film against 200 leucocytes. Estimation of the parasite density by light microscopy was expressed as the number of parasites per microlitre of blood^15^. *Plasmodium* species was identified on the thin blood smear.

### Genomic DNA extraction

For each participant, a Dried Blood Spot (DBS) was prepared and transported at Centre for Research in Infectious Diseases (CRID) laboratory. DNA was extracted from suspected false-negative (negative by RDTs and positive by microscopy) and false positive (positive by RDTs and negative by microscopy) blood samples using the saponin/Chelex method, as previously described^16^. DNA was also extracted from six true positive samples (positive to both microscopy and RDTs) and two true negative samples (negative to both microscopy and RDTs), which were used as positive and negative controls, respectively. In brief, blood spots were incubated overnight at 4 °C in 1 ml of 0.5% saponin in phosphate-buffered saline (PBS). The spots were washed for 30 min in PBS at 4 °C, transferred to new tubes containing 200 µl of 5% Chelex100^®^ (Bio-Rad Laboratories CA), and mixed for 30 s. The tubes were then heated at 95 °C (in a water bath) for 15 min to elute the DNA, vortexed, and centrifuged at 13,500 rpm for 5 min. The supernatants (180 µl) were transferred to new tubes for a second centrifugation. From this second centrifugation, 130 µl of supernatant was removed and transferred to a new tube.

### Molecular detection of malaria parasites by real-time quantitative PCR

While microscopy is referred to as the reference, qPCR is acknowledged as more sensitive and was used for validation, especially when results obtained by tests were discordant. The *18S small subunit ribosomal RNA* gene *(18SRNA)* was amplified for the molecular detection of *P. falciparum* and non-*falciparum* (OVM+: *P. ovale*,* P. vivax*,* and P. malariae*) infections. The experiment was performed using the Taqman assay as previously described, with slight modifications^17^. DNA amplification was carried out in a total volume of 10 µL of mix, containing 0.3 µL and 0.2 µL of Falcip+ (5‘TCT-GAA-TAC-GAA-TGT-C3’) and OVM+ (5‘-CTG-AAT-ACA-AAT-GCC-3’) probes, respectively, 0.8 µL of primers PlasF (5‘GCT-TAG-TTA-CGA-TTA-ATA-GGA-GTA-GCT-TG3’) and PlasR (5‘GAA-AAT-CTA-AGA-ATT-TCA-CCT-CTG-ACA 3’), 5 µL of sensimix, and 1 µL of DNA. PCR conditions were as follows: 10 min at 95 °C followed by 40 cycles of 92 °C for 15 s and 60 °C for 1 min.

### Data analysis

Raw data were entered into Microsoft Excel 2019 and exported to Rstudio and SPSS V23 for statistical analysis. In Rstudio, sensitivity, specificity, positive and negative predictive values (PPV and NPV), positive and negative likelihood ratio (LH + and LH-) ratios, and accuracy were calculated to assess the performance of the RDT compared with microscopy and qPCR using epiR package. Cohen’s Kappa (**k**) and Gwet’s statistics (**AC1**) were calculated by the irrCAC package^18^ to measure the agreement between the different assays (RDT, microscopy and qPCR). Kapa value of < 0.59, 0.6–0.79, 0.8–0.9 and > 0.9 were interpreted as weak, moderate, strong and almost perfect agreement, respectively. The Gwet’s coefficient was used when the “kappa paradox” (low Kappa values despite high observed agreement) was observed and was interpreted in a similar way to Cohen’s Kapa value.

With SPSS V23, differences between point estimates (malaria prevalence, parasite density) according to socio-demographic variables (age group, sex) or other collected variables (temperature, test sensitivity) were studied using the Chi square. A p-value less than 0.05 was considered significant.

## Results

### Sociodemographic and clinical characteristics of study participants

**Fig. 1:**
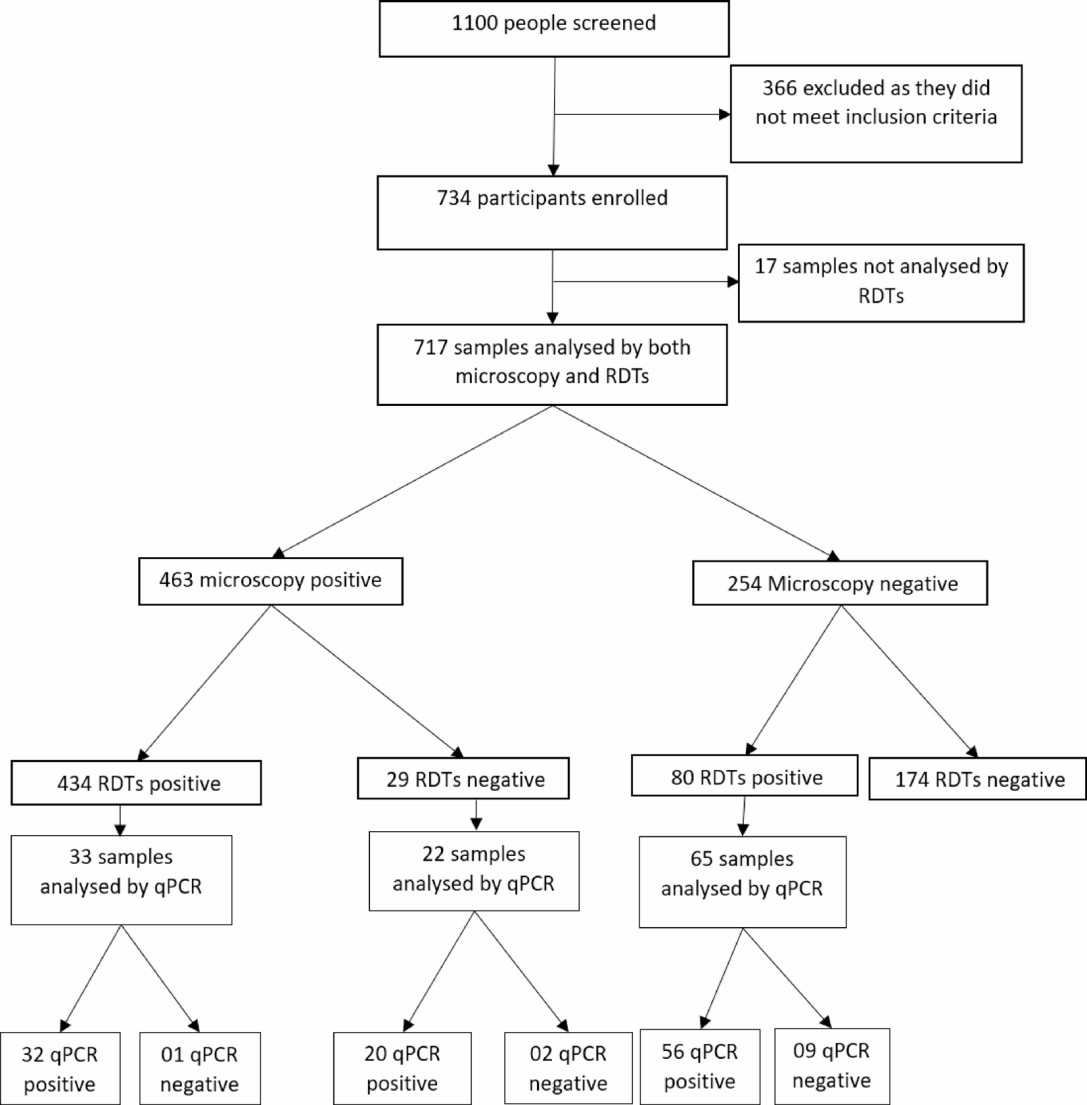
Study flow diagram showing patient enrolment and diagnostics performed.

A total of 717 participants were enrolled in the study, among which 247 were recruited in the community and 470 at primary schools (Fig. 1). The sex of 617 participants was successfully recorded with female being more represented (54.78%). The age group of 5 to 14 years was the most abundant (69.19%), while children under five years consisted of 10.81% of the study population. Only a few (3.90%) participants had fever, which is the primary sign of malaria (Table 1).

**Table 1:** Malaria infection parameters in the study populations.

						RDT					Microscopy		
**Variables**				**Effective**		**Prevalence** **(%)**	**Confidence** **Interval**	**P value**	**Effective**		**Prevalence** **(%)**	**Confidence** **interval**	**P value**
**Villages**													
	Emana			89		65.2%	55.1% −75.3%	0.055	89		58.4%	48.0% −68.9%	0.070
	Nkol-Feb			98		52.0%	42.0% −62.1%		98		46.9%	36.9% −57.0%	
	Nkoloudou			60		46.7%	33.7% −59.7%		60		40.0%	27.2% −52.8%	
**Total**				**247**		**55.5%**	**49.0% − 61.8%**		**247**		**49.4%**	**43.0% − 55.80%**	
**Schools**													
	Ebougsi			97		86.60%	79.7% − 93.5%	0.024	112		**82.10%**	74.9% − 82.3%	0.017
	Lengon			97		81.4%	73.6% − 89.3%		97		73.20%	64.2% −82.2%	
	Elig-Onana			35		94.30%	86.2% − 100%		35		80.00%	66.1% − 93.1%	
	Mva’a 1			150		76.0%	69.1% − 89.2%		152		71.1%	63.8% −78.3%	
	Zamengoue			91		73.6%	64.4%- 82.9%		91		61.50%	51.4% − 71.7%	
**Total**				**470**		**80.2%**	**76.3% − 83.7%**		**487**		**73.1%**	**69.0% − 77.1%**	
**Sex**													
	Female			338		65.1%	60.0% −70.2%	0.011	338		59.8%	54.5% −65.0%	0.236
	Male			279		74.6%	69.4% −79.7%		281		64.4%	58.8% −70.0%	
**Total**				**617**		**69.4%**	**65.6% − 73.0%**		**619**		**61.9%**	**57.9% − 65.7%**	
**Age**													
	< 5			67		74.6%	63.9% − 89.3%	< 0.001	67		61.2%	49.2% −73.2%	< 0.001
	[5–10[			244		77.5%	72.2% − 82.7%		244		73.0%	67.3% −78.6%	
	[10–15[			185		81.6%	76.0% − 87.3%		187		71.7%	65.1% −78.2%	
	[15–20[			21		61.9%	39.3% −84.6%		21		57.1%	34.1% − 80.2%	
	[20–30[			15		26.7%	1.3–52.0%		15		26.7%	1.3% −52.0%	
	[30–40[			14		35.7%	7.0–64.4%		14		21.4%	0.0% −46.0%	
	[40–50[			17		23.5%	1.0% – 46%		17		23.5%	0.0% − 46.0%	
	> 50			57		24.6%	13.0% − 36.1%		57		15.8%	0.6% − 25.6%	
**Total**				**620**		**69.3%**	**65.6% − 73.0%**		**622**		**61.9%**	57.9% − 65.7%	
**Temperature**													
	Normal			689		71.8%	68.5% − 75.2%	0.640	706		64.7%	61.2% − 68.3%	0.548
	Fever			28		67.9%	49.4% − 86.3%		28		71.4%	53.6% −89.3%	
**Total**				**717**		**71.7%**	**68.4% − 75.0%**		**734**		**64.9%**	**61.1% − 68.1%**	

### Prevalence of malaria infection and *Plasmodium* species

The prevalence of *Plasmodium* infection detected by microscopy in the study population was 64.9% (IC: 61.1%- 68.1%). Of the 717 participants tested simultaneously by microscopy and RDT, the infection rate detected by the RDT was 71.7% (IC: 68.4% − 75.0%) and was significantly higher (*p* < 0.001) than that observed by microscopy (*P* = 64.6%; IC: 61.1% 68.1%) (Fig. 1).

In communities, almost half of the participants was infected (*P* = 49.4%) with the population of Nkol-feb having the higher infection rate (*P* = 46.9%; CI: 36.9%- 57.0%) while almost 3/4 (*P* = 72.5%) of the children recruited in schools had *Plasmodium* infection. Students from Ebougsi primary school had the higher infection rate (*P* = 82.1%; CI: 79.1%- 89.3%) (Table 1).

The majority of infections were caused only by *P. falciparum* (372/477). Twelve cases of mono-infection by *P. malariae* and 93 cases of mixed infection by the two parasites species were observed. Asexual parasite densities in blood ranged from 40 to 250,440 parasites/µL for *P. falciparum* (median:1040; interquartile range: Q1 = 440; Q3 = 2540) and from 40 to 5760 parasites/µL for *P. malariae* (median:200; interquartile range: Q1 = 120; Q3 = 480).

### Performance of the Abbott Bioline Malaria Ag P.f/Pan RDT compared to microscopy

A total of 717 blood samples were tested in parallel by microscopy and RDT. With microscopy as reference, the RDT showed a sensitivity of 93.73 (CI:91.15%- 95.77%) for the detection of *P. falciparum* infection as only 29/463 (6.26%) samples detected as positive by microscopy were negative by RDT. However, its specificity was considerably reduced (Sp: 68.50, CI:62.40% − 74.17%) due to the high rate of FP (31.50%) samples observed. This also affected the positive predictive value of the test (PPV = 84.44%; CI: 81.01% − 87.46%). People with infection by *P. falciparum* were two times more likely to have positive RDT result than those without the infection (LR + = 2.97; CI: 2.47–3.57). There were low probabilities for the RDT to give a negative result in people with the infection (LR-= 0.09; CI: 0.06–0.13). The rapid test demonstrated a substantial agreement (k = 0.652; CI:0.593–0.710; *p* < 0.001) with microscopy, suggesting that the Abbott Bioline RDT is likely to be of good quality (Table 2).

**Table 2:** Performance of the Abbott Bioline Malaria Ag P.f/Pan RDT with microscopy as reference.

Performance parameters	Values (CI)%	Reference***
Sensitivity*	**93.734 (91.15–95.77) %**	99.70%
Sensitivity**	**17.71 (14.34–21.50) %**	95.50%
**Specificity**	68.50 (62.40–74.17) %	99.50%
**Positive predictive value**	84.44(81.01–87.46) %	-
**Negative predictive value**	85.71(80.13–90.22) %	-
**LR+**	21.97(2.47–3.57)	
**LR-**	0.09 (0.06–0.13)	
**Accuracy**	0.85 (0.82–0.87)	
**Kappa**	0.652(0.593–0.710)	

*Sensitivity of the test for the detection of *P. falciparum*; **sensitivity of the test for the detection of plasmodium species other than falciparum; *** test performance values provided by the manufacturer; sensitivity = TP/FN + TP; specificity = TN/TN + FP; likelihood ratio for a positive test (LR+) = sensitivity/(1 – specificity); likelihood ratio for a Negative test (LR-)= (1 − sensitivity)/specificity). .

A low sensitivity (Se = 17.71%; CI: 14.34% −21.50%) and specificity (Sp = 68.50%; CI: 62.40% − 74.17%) of the test was observed for the detection of species other than *P. falciparum*. The predictive values of the test for the Pan LDH were also low [(PPV = 50.62%; CI: 42.66% − 58.55%); NPV = 31.35% (27.51% − 35.39%)], as well as the positive and negative likelihood ratios [(LR + = 0.562; CI: 0.430–0.734); LR-= 1.201 (1.094–1.318)].

### Performance of the RDT according to parasite density

An association between RDT sensitivity and parasite density was observed (X^2^ = 25.083; *p* = 0.001). Actually, a 23.5% decrease in sensitivity of the RDT was observed at parasite densities less than 50 parasites/µl of blood. Although a small proportion of FN were found at parasite densities of up to 5000 parasites/µl of blood, the test showed a maximum sensitivity (100%) for very high parasite densities (over 5000 parasites/µl of blood) (Table 3).

**Table 3:** Sensitivity of Abbott Bioline Malaria Ag P.f/Pan RDT according to parasite density.

Number of parasites/uL of blood	Infection by *P*. *falciparum* detected by microscopy	Positive to PfHRP2	Sensitivity (FN*)	Confidence Interval	*P*. value	Reference **
**1–50** **51–100** **101–500**	17 4 108	13 4 96	76.5% (4) 100.00% (0) 88.9% (12)	54.0% − 99.0% NA 82.9%- 94.9%	0.001	93.8% 100.0% 100.0%
**501–1000**	93	90	96.8% (3)	93.1% − 100%		100.0%
**1001–5000**	163	158	96.9% (5)	94.3% − 99.6%		100.0%
**> 5000**	69	69	100.00% (0)	NA		100.0%
**Total**	454	430	94.7% (24)	92.6% − 96.8%		99.70%

*FN = false negative; ** reference values of the manufacturer.

### Performance of RDT and microscopy compared to qPCR

A subset of 120 blood samples (22 FN, 65 FP and 33 TP) were analysed by qPCR to look at their infection status. With qPCR as the gold standard, the RDT showed high sensitivity of 81.48% (CI: 73.57% – 88.14%) but low specificity (Sp = 16.67%; CI: 2.09%- 48.41%). By contrast microscopy showed low sensitivity (Se: 48.14%; CI: 38.43% − 57.97%) but high specificity (Sp: 75.00%; 42.81% − 94.51%). In fact, 20/22 (90.90%) of the FN samples were positive by qPCR, with *P. falciparum* being the main specie involved. However, the Pan LDH protein was not detected by the RDT in any of those samples. Interestingly, 56/65 (86.15%) samples considered false positive were confirmed positive by qPCR, with *P. falciparum* being the main species. All but one of them were from asymptomatic participants. Few cases of co-infection with other *Plasmodium* species (OVM+) (06/56) were observed. Only one of the 33 true positive samples tested was found negative by qPCR. The RDT showed a high agreement with (AC1 = 0.715; IC: 0.617–0.813) qPCR than microscopy (AC1 = 0.317; IC: 0.246–0.497) (Table 4).

**Table 4:** Performance of Abbott Bioline Malaria Ag P.f/Pan RDT and microscopy compared to qPCR.

Performance parameters	RDT	Microscopy
Sensitivity*	81.48 (73.57–88.14) %	48.14 (38.43–57.97) %
Specificity*	16.67 (2.09–48.41) %	75.00 (42.81–94.51) %
Positive predictive value*	89.69 (82.03–95.00) %	94.55 (84.88–98.86) %
Negative predictive value*	9.09% (1.12–29.16) %	13.85(6.53–24.66) %
LR+*	0.96 (0.7475–1.2790)	1.92(0.71–5.23)
LR-*	1.03 (0.29–4.18)	0.69 (0.48–1.00)
Accuracy*	0.75 (0.66–0.82)	0.50 (0.41–0.60)
Gwet’s AC1*	0.715 (0.617–0.813)	0.317 (0.246–0.497)

* 95% confidence of interval.

## Discussion

This study determined the prevalence of malaria infection in people living in two malaria endemic rural areas of Central Cameroon and evaluated the diagnostic performance of the Abbott Bioline Malaria Ag P.f/Pan RDT which is extensively used for malaria diagnosis in the communities and health facilities with limited access to microscopy.

Based on the microscopy, which is the gold standard for malaria diagnosis, more than half (65.0%) of the tested people harboured *Plasmodium* infection, although almost all (96.1%) were asymptomatic. The trend of *Plasmodium* parasite carriage observed indicates that the Centre region of Cameroon, remains holo-endemic despite the efforts made to curb the disease incidence^19–21^. Due to its climate, vegetation cover and hydrography, this region offers favourable conditions for the development of the major vectors (*An. gambiae*,* An. coluzzii*, and *An. funestus*) of *Plasmodium* throughout the year, which maintain perennial malaria transmission. In addition, other factors such as the non-use or misuse of LLINs or their reduced effectiveness due to their physical deterioration and/or the development of insecticide resistance in vectors can also contribute to the persistence of such a high level of malaria transmission^3^. It is worth mentioning that the last campaign of mass distribution of LLINs took place more than three years prior the present study. Thus, it could be possible that LLINs coverage has dropped and/or LLINs are damaged or have lost their bio-efficacy, further explaining the high infection rate observed among school-aged children. Moreover, asymptomatic carriers of *Plasmodium* parasites represent an infectious reservoir that maintains malaria transmission. The asymptomatic nature of infection in the vast majority of cases is thought to be linked to the fact that in areas of high malaria transmission, repeated exposure to the bites of infected mosquitoes makes the host’s immune system better able to control the density of parasites, thus preventing or delaying the onset of clinical symptoms^22^.

The Abbott Bioline Malaria Ag P.f/Pan RDT is extensively used for malaria case management in Cameroon. This RDT showed good sensitivity (93.73%) but low specificity (68.50%) to detect *P. falciparum* infection compared to microscopy. Furthermore, the qPCR also confirmed the high sensitivity of the RDT as 56 of 65 (86.15%) falsely considered positive samples by microscopy were confirmed positive by this technique. Although our values were lower than manufacturer’s reference ones (Se: 99.70%; Sp: 99.50%), the Abbott Bioline test remained in line with the WHO standard (≥ 75% at 200 parasites/µl)^23–25^, thus supporting its use as a reliable diagnostic tool for case management of uncomplicated malaria in communities, especially when microscopy is not available. Nevertheless, similar studies should be conducted in symptomatic patients, or in low malaria transmission area as in West Cameroon, as well as in seasonal transmission areas in the North, to better capture the performance of this RDT in the context of different malaria endemic settings. Actually, previous research works have reported that the performance of RDTs in symptomatic cases or in high malaria transmission settings tends to be higher due to the presence of higher parasite densities, which makes detection easier. In contrast, the performance in asymptomatic cases or in low endemic settings is often lower due to the challenges associated with detecting low parasite densities^26–28^.

The non-negligible proportion of false negative results observed in this study calls for routine and active surveillance of the performance of this RDT across the country. False negative diagnosis can have harmful consequences, as it prevents the decision for the management of true cases of infection with antimalarial drugs. Two consequences can result from this situation: (i) the persistence of the parasite reservoir in a community, which favours new contamination via *Anopheles* vectors and contributes to an increase in the morbidity of the disease^29^ and/or (ii) the worsening of cases from uncomplicated to severe malaria with the risk for an increase in mortality in the absence of appropriate treatment, particularly in children aged under five considered more vulnerable to the infection. It therefore remains important, whenever possible, to confirm any RDT-based diagnosis with microscopy. One of the most important causes of false-negative results obtained by RDTs that target the PfHRP2 protein is the deletion of the *pfhrp2* gene that codes for its synthesis, hence the developement of tests that can simultaneously detect both *pfrrp2* and LDH (Pan) antigens, to improve diagnosis accuracy^ 30^. In this study, the Pan (LDH) band test struggled to give appropriate results for people infected by species other than *P. falciparum* compared to non-infected ones (low LR + = 0.562 and LR-=1.201). In addition, the Pan test line did not appear in any samples that were positive by microscopy and negative for *pfhrp2* detection, highlighting the poor ability of the Abbott Bioline Malaria Ag P.f/Pan RDT to detect suspected cases of *Pfhrp2* deletion. Given that strains of *P. falciparum* with a *pfhrp2* deletions have previously been reported in Cameroon^31,32^, it will therefore be interesting to conduct appropriate investigation of *pfhrp2* and *pfhrp3* gene deletions using sequencing methods, to elucidate its contribution to lower performance scores of the RDT.

Another significant challenge associated with RDTs is misdiagnosis due to false-positive results. There are several consequences associated with false positive malaria results including a delay for appropriate care for other causes of fever such as viral or bacterial infections, an administration of unnecessary antimalarial treatments that can increase healthcare cost and contribute to the growing threat of drug resistance or to an overestimation of malaria cases affecting statistics important for resource allocation, policy formulation, and health program management^30^. In this study, the rate of false positive results (positive by RDTs but negative by microscopy) was 15.56% and was above the limit recommended by WHO (10%)^23–25^. Likely, most FP results observed were due to submicroscopic infections rather than persistence of *pfhrp2* antigen in the bloodstream, since qPCR analysis of a subset of 65 FP samples confirmed that 86.15% of them were positive. The detection threshold for microscopy, when performed by expert microscopists using good quality equipment, is estimated at 4–20 parasites/µL of blood^11^. However, cases of sub-microscopic infection (very low parasite density), most often observed in asymptomatic people in the community, generally go undetected by this technique. As observed in this study and previous ones^33–36^, the higher sensibility of RDTs compared to microscopy, due to their ability to detect sub-microscopic infections, represents an advantage for the management of uncomplicated malaria in the community.

## Conclusion

This study highlights the high performance of the Abbott Bioline Malaria Ag P.f/Pan RDT in detecting *Plasmodium* infection in asymptomatic peoples living in the high malaria endemic setting of rural forested Central Cameroon, making it a reliable diagnostic tool for malaria case management. However, the non-negligibly proportion of false-negative RDT results obtained call for routine and active surveillance of the performance of this RDT in different endemic settings across the country. Quantifying parasite density by molecular methods and investigating *Pfhrp2/3* gene deletions in this study would have provided more insights on the potential causes of false negative results. These information are crucial for understanding the limitations of studied RDT and improving malaria diagnosis and control.

## Data Availability

Data are available from the corresponding author on reasonable request.
